# The Effects of the Maternal Health Improvement Project in the Louga Region of Senegal

**DOI:** 10.3390/ijerph19010396

**Published:** 2021-12-30

**Authors:** Babacar Ndiaye, Louis Thiam, Gahee Ham, Yunsung Choi, Eunmi Lee, Kilho Kang, Youngran Yang

**Affiliations:** 1 Plan International Senegal, Louga 21121, Senegal; babacarndiaye47@yahoo.fr (B.N.); Louis.THIAM@plan-international.org (L.T.); 2 Plan International Korea, Seongnam 13494, Korea; hamgh@plankorea.or.kr (G.H.); choiys@plankorea.or.kr (Y.C.); 3 College of Nursing, Jeonbuk National University, Jeonju 54896, Korea; diazepam4@jbnu.ac.kr; 4 VF Partners, Seoul 06732, Korea; khkang@vfp.co.kr; 5 College of Nursing, Research Institute of Nursing Science, The Center for Sustainable Development, Jeonbuk National University, Jeonju 54896, Korea

**Keywords:** antenatal care, contraception, danger signs of pregnancy, maternal health, maternal mortality, Senegal, IEC activities

## Abstract

Maternal mortality remains a major global health challenge in sub-Saharan Africa. Senegal is one of the countries in the region that lagged behind in reaching the Millennium Development Goal 5, the deadline of which passed in 2015. The objective of this study was to assess the effects of information, education, and communication (IEC) activities conducted in Louga, Senegal. Community groups and facilitators conducted IEC campaigns, home visits, and various awareness-raising activities. This study used secondary data as part of the baseline and mid-term evaluations. Participants included women and men who had one or more children under five years of age. It was found that the level of awareness of at least three danger signs of pregnancy recognised by men significantly increased, and husbands/partners more frequently accompanied their wives during antenatal care in 2019 than in 2018. Women’s empowerment improved significantly in terms of women making their own health decisions, joining community decision-making associations or groups, and using contraception. This project indicates that policies and programs are needed to increase men’s involvement and empower women to further women’s reproductive health to achieve the Sustainable Development Goal 3 and reduce maternal mortality in Senegal.

## 1. Introduction

According to the latest World Health Organization figures, every day in 2017, approximately 810 women died from preventable causes during pregnancy or childbirth. Almost all of these deaths (94%) occurred in low- and lower-middle-income countries. Sub-Saharan Africa is the continent most affected by such deaths. The high number of maternal deaths result from inequalities in opportunities to use quality health services and represents the gap between rich and poor countries. The maternal mortality rate (MMR) in low-income countries in 2017 was 462 per 100,000 live births versus 11 per 100,000 live births in high-income countries [1]. These overwhelming figures highlight maternal mortality as an urgent public health issue in developing countries. To fight this issue, one of the Millennium Development Goals (MDG5) was to reduce maternal mortality by three-fourths by 2015, making low-risk maternity a priority [2]. Taking fresh impetus from the MDGs, in 2015, the UN adopted a new sustainable development agenda, which focused on 17 sustainable development goals (SDGs) to substitute the MDGs. Under the SDG agenda, SDG3.1 has led to a new maternal health transformation initiative to end preventable maternal mortality so that the global maternal mortality rate can decrease to below 70 per 100,000 live births by 2030 [3].

Senegal lagged behind in reaching MDG5, a goal which had set 170 maternal deaths per 100,000 live births as its target. To achieve the SDGs and in line with Senegal’s national policy based on the National Health Development Plan (PNDS), the country has made considerable efforts by launching several programs to reduce the burden of maternal, neonatal, and infant mortality. To meet these challenges, in addition to the PNDS, Senegal has defined a health policy that stresses on national strategic programs, including the National Family Planning Action Plan 2012–2015 [4] and the National Strategic Plan for Child Survival 2007–2015 [5].

According to a systematic review of nine low- and middle-income countries, programs engaging men during pregnancy, childbirth, and infancy resulted in improvement of antenatal care (ANC) attendance, facility birth, complications preparedness, and maternal nutrition, as well as male partner support for women and couple communication, and joint decision-making [6]. Many of these programs used the theory of change as a framework to coherently describe their project activity components, intended outcomes, and long-term goals. The Maternal and Child Health (MCH) program implemented by the Ministry of Public Health of the Democratic Republic of Congo (DRC) improved the ANC utilisation by more than four times at the project site compared to the control area (odds ratio 2.280, 95% CI: 1.133–3.902). The project included an educational program to increase the awareness of ANC through home visits, radio broadcasting, and signboards [7]. In a study of a community-based intervention program conducted in the Democratic Republic of Zambia, the provision of education on pregnancy and childbirth management increased the knowledge of women regarding ‘pregnant women must receive ANC in early pregnancy’ and ‘three or more dangerous risk signs of pregnancy’ by 24.7% and 7.1%, respectively, compared to before the educational intervention. Furthermore, the intervention program reported that the delivery rate at medical facilities and the proportion of women who used contraception after childbirth increased by 25.7% and 10.6%, respectively [8]. A study conducted in Ethiopia’s Tigray region used small group education with the intervention group at the village level and educated them using bulletin boards to report higher increased rates of contraceptive use, prenatal management, facility delivery, and postpartum medical check-ups compared to the control group which was provided information using local radio [9].

Owing to effective interventions at both the clinical and community levels, Senegal has become one of the few countries where significant progress is being made in maternal and newborn health. The maternal mortality ratio decreased from 510 deaths per 100,000 live births in 1992 (District Health Information System DHS II) to 315 in 2015 (ANSD) [10]. However, the maternal mortality rate remains a concern, particularly in regions such as Louga (400 deaths per 100,000 live births in 2015), which are wide with remote and inaccessible health facilities. To overcome these challenges, Plan International Senegal, in partnership with Plan International Korea, launched a project to reduce maternal and child mortality in the health districts of Sakal, Keur Momar Sarr, and Coki in the Louga region. The goal of the three-year project ‘Better Maternal Health’ was to contribute to the reduction of maternal and child mortality in the region by improving the quality of health services, raising community awareness on reproductive health, and improving access to health services.

In the first year of the project, between July and September 2018, a baseline survey was conducted in the three target health districts. Subsequent to the baseline survey, after one year of implementation the mid-term evaluation of the performance measurement framework (PMF) occurred in June 2019 to collect relevant information that could help identify possible corrections applicable to the process, to optimise the implementation of activities, better meet the needs of the target populations, and identify relevant interventions for scaling up. This study used secondary data as a part of the baseline survey and mid-term evaluation to determine the effects of maternal health improvement by focusing on the changes in awareness of the danger signs of pregnancy, husbands accompanying their wives to ANC visits, women’s acknowledgement of having received the support of the husband during pregnancy, delivery at appropriate health facilities, and women’s decision-making about health, community activities, and contraception.

## 2. Materials and Methods

### 2.1. Research Design

This study used a one-group pretest-posttest design, to compare the before and after stages of the project implementation and determine the project’s effect on maternal health in Louga. 

### 2.2. Study Area

**Figure 1:**
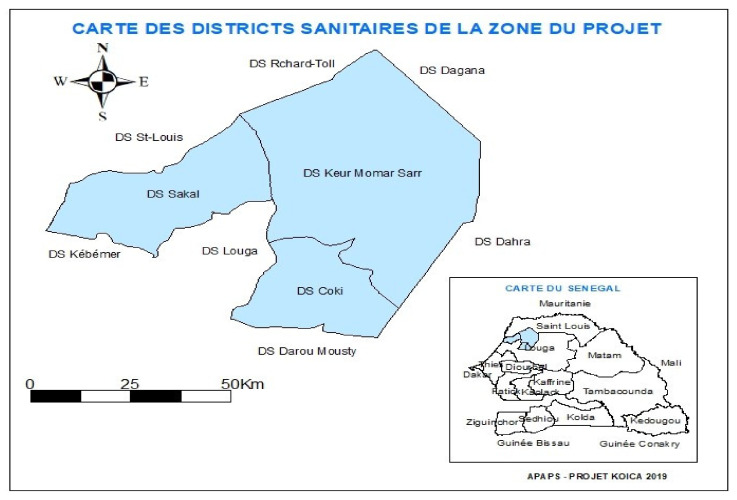
Area of the study.

The data collection was carried out in the Louga region, particularly in the communes of the project’s three target health districts, Coki, Keur Momar Sarr, and Sakal (Figure 1).

### 2.3. Study Participants

The study participants were women of reproductive age (WRA) aged 15 to 49 years with at least one child aged under 5 years, and men aged 15 to 59 with a partner and with at least one child aged under 5 years. The participants were residents of the districts of Coki, Keur Momar Sarr, or Sakal. The WRA represented 24.2% of the total population in the study area. More than one third of the WRA (young people aged 15–19 years and adults aged 40–49 years) were at a high risk of maternal mortality.

### 2.4. Sampling

A random sample, representative of the health district, was drawn directly from the sampling frame using data from the 2013 General Census of Population, Habitat, Agriculture, and Livestock (RGPHAE 2013). First, each of the three health districts was subdivided into clusters [here, the RGPHAE Census Districts (RDs)] from which a random sample of census districts was drawn, followed by a subtracted draw within each census district sample. Finally, in each sample household, the WRA with at least one child aged under five years and the man of the household whose partner had a child under five years were interviewed. Thus, a 3-degree sampling method was used with the WRA and men as survey units. The optimal sample size for each stratum was determined using the Schwartz formula, with an accuracy of 5% and a confidence level of 95% based on the key indicator of the study, which represents the proportion of WRA who are users of modern contraception:

(1) $$n=\frac{t^{2}p(1-p)}{e^{2}}$$

where

*n* = optimal sample size to be sought;
*t* = standard value (1.96) corresponding to the expected confidence level;
*p* = estimated value of the selected indicator (proportion of WRA users of modern contraception;
*e* = degree of accuracy of margin of error at 5% (typical value: 0.05).

According to the outcomes of the continuous Demographic and Health Survey (DHS) of 2016, the proportion of WRA using modern methods of contraception in rural areas was 17.2%. Calculations using the Schwartz formula resulted in a survey size of 219 survey units as the optimal size of the WRA sample to be surveyed by stratum. This last figure, rounded to 220, was used as the size of the WRA subsamples to be surveyed by the health district. This resulted in a final size of 250 WRA samples per stratum, that is, 500 WRA for the two strata. No adjustment was made to consider the cluster effect and losses due to the small proportion of households that would be selected per cluster (or DR) and the high potential for replacements. This resulted in an optimal size of 220 WRA samples per health district, adding up to 660 WRA for all three districts. The same applied to the men who were surveyed in the same household as the WRA.

### 2.5. Project Intervention

Thirty-seven facilitators in the community groups (community-based organisations (CBOs)) and 22 project assistants were a part of the study. The facilitators conducted information, education, and communication (IEC) campaigns, home visits, and talks with the villagers. The project assistants supported the monitoring and supervision of these activities. Service quality management, which included sharing suggestions of the facilitator and project assistant evaluations conducted in the first quarter, was carried out.

A total of 1520 IEC campaigns, 9198 village-based informal talks, and 10,016 home visits were conducted, and a total of 292,461 (male 71,308/female 221,153) participated in these programs. The IEC campaigns were conducted with 30 or more people as a large group, and the facilitators used IEC materials for education. Village-based informal talks were carried out with a limited group of 15 to 25 people, in which facilitators interacted with participants during sessions, enabling participants to ask clarifying questions. Home visits were conducted for interpersonal awareness-raising activities in which the facilitators visited the targeted persons. During home visits, all health issues, including taboo topics, could be discussed with the facilitator and the facilitator also referred the pregnant women to the health facility. Through these three measures, various awareness-raising activities were carried out for residents, such as the importance of prenatal management, postpartum management, and male childbirth support. Details of the programs’ contents included the following: maternal and child health knowledge and awareness of beneficiary populations regarding pregnancy-related risks, the number of ANC visits required during pregnancy, and the benefits of skilled delivery. The IEC activities addressed the attitudes and behaviours of the beneficiary populations regarding maternal health and gender equity issues, such as male partners’ assistance to WRA before, during, and after delivery, and the level of support provided by the male partner in the female partner’s decision-making processes.

A radio program on reproductive health and the benefits of getting medical insurance was conducted by a midwife on three radio rations. The program was intended to improve the awareness of those who have the right to make decisions at home, such as the elderly, but who have difficulty participating in awareness-raising activities through CBOs. During the project year, 251 radio shows were carried out. The facilitators conducted five meetings with the coordinators of the CBOs to coordinate and discuss activity progress, review evidence, follow regulations, activity priority, and activity plan.

### 2.6. Data Collection

Data collection was carried out simultaneously in the three health districts by 15 field staff. The staff members were split into three teams of four enumerators with a supervisor. For comparability of data, the data collection of the second-year study adopted the same techniques and tools as the baseline study. The survey data were collected from 7 August to 20 August 2018, for baseline and from 29 September to 10 October 2019 for the second year. The survey of socio-economic, demographic, and health data was carried out in the three health districts. In each sample census district of the household survey, the team supervisor contacted the local authorities to inform them about the purpose of the survey, the targets to be interviewed, and the expected practical arrangements. Once in a selected household, one of the enumerators proceeded to first inventory the household members to determine eligible participants (men and women). The enumerator then administered the questionnaire to eligible men and women after obtaining informed consent. The household survey was conducted in an electronic format (tablets). Once the questionnaires were completed, and checked by the supervisor, they were transferred daily to a server set up by the study agency. To ensure quality of the data, a second check for completeness and consistency was carried out by the management team.

### 2.7. Measures

‘Awareness of the danger signs of pregnancy’ was measured as being aware of at least three pregnancy danger signs. The participants were asked two questions, ‘Do you know any danger signs related to pregnancy?’ ‘If yes, what are the danger signs related to pregnancy that you can quote?’ (open questions). If the participants quoted more than three out of the nine signs (lack of foetal activity; convulsion; intense fever; severe, permanent headache/blurred vision; oedema of the face, hands, and legs; paleness; water loss; vaginal bleeding; and intense stomach pain), it was coded as having awareness of the danger signs of pregnancy.

‘Husbands accompanying their wives during ANC visits’ was measured by the question, ‘Who accompanies you during ANC visits?’ and among the options were husband/partner, mother, brother/sister, aunt, mother-in-law, sister-in-law, co-wife, friend, relay/matron, or nobody. ‘Did your husband/partner support you during your last pregnancy?’ Yes/No), was asked to assess the women’s acknowledgement of having received support from their husband/partner during pregnancy. Delivery at appropriate health facilities included the options: clinic, health centre, health hut, health post, and hospital to the question ‘Where did you give birth to your last child?’. Women’s empowerment was measured by three aspects: making one’s own health decisions (‘Who generally makes decisions about your health care?’ with the options only by wife, both husband and wife, husband/partner, or mother-in-law), joining a community decision-making association or group (‘Have you joined a community decision-making association or group?’ Yes/No), and disagreement with husband about the use of contraception (‘Does your husband/partner forbid you to use a method of contraception?’ Yes/No).

General characteristics were also obtained, including age, education level, marital status, ethnic group, and religion.

### 2.8. Ethical Considerations

Before data collection, the study was approved by the National Health Research Ethics Committee (CNERS) (approval number for the baseline survey: Ethical and scientific notice N ° 000084/MSAS/DPRS/CNERS dated 31 August 2018; for the midterm survey: N ° 000165/MSAS/DPRS/CNERS dated 6 September 2019). In compliance with the agreement, participation in the survey was anonymous and voluntary. Informed consent was obtained from all participants. In advance, they were informed of their right to refrain from participating or to withdraw from the survey whenever they wished without fear of reprisal. In the event of an agreement to participate in the study, the enumerator signed a consent form and gave a copy to the respondent.

### 2.9. Statistical Analysis

Collected data were analysed using descriptive statistics, including frequency and percentage, and a chi-square test or ANOVA was used to assess the effectiveness of the IEC activities before and after the intervention in the study areas. IBM SPSS Statistics for Windows, Version 21.0; IBM Corp., Armonk, NY, USA was used for data analysis.

## 3. Results

### 3.1. Characteristics of the Participants

The age structure of the women remained relatively stable and was marked by youth. More than a third of the women surveyed (39.0%) in 2019 were under 25 years of age (compared to 34.8% in 2018). The percentage of 40-year-olds and above was still very low (6.7% in 2018 versus 6.7% in 2019). The average age was 26 years in 2019 compared to 28 years in 2018. The percentage of out-of-school women was 53.3% in 2019 and 65.2% in 2018. The percentage of respondents who had reached secondary school or higher slightly increased, from 2.0% in 2018 to 7.1% in 2019. The distribution of samples by marital status changed, showing an increase while maintaining the same structure. Married polygamous couples decreased from 39.1% in 2018 to 36.6% in 2019, but the difference was not significant. The predominance of Ouolofs and Poulars still marked the structure by ethnic group, followed by Moors to a lesser extent. Almost all of the women surveyed were Muslim, and almost half reported that the health facility was far from their home (Table 1).

The average age (40 years) of men remained the same in both surveys as did the age group structure. The level of education was still dominated by Koranic education (58.3% in 2019 against 71.1% in 2018). However, the numbers of those who had attended formal school was higher in 2019, with 17.9% of the respondents compared to 10.1% in 2018. Moreover, similar to women, almost all the men interviewed during the mid-term evaluation were married and Muslim. The same applied to their ethnic group, which was, in most cases, Ouolof or Poular (Table 2). Overall, despite slight differences in the socio-demographic characteristics of respondents (male and female) in both surveys, the outcomes are comparable because a few of the chi-square tests between 2018 and 2019 were statistically significant (educational level and level of proximity of health facility among women).

### 3.2. Awareness of Danger Signs of Pregnancy

The level of awareness of at least three danger signs of pregnancy indicated awareness of men significantly increased between 2018 and 2019: 14.2% against 20.9% (*p* = 0.029) (Table 3). The best-known signs for men in 2019 were severe stomach pain, followed by vaginal bleeding and intense fever, while in 2018, intense fever was mentioned first. However, awareness among women decreased slightly (45.8% in 2018 compared to 41.5% in 2019).

### 3.3. Husbands Accompany Their Wives during ANC Visits

In 2019, husbands/partners more frequently accompanied their wives during ANC visits than in 2018: 22.3% compared to 14.7% (*p* = 0.010). The main reasons for non-support in 2019 remained the same as in 2018—absence of the husband/partner, respect of the customs, or preoccupation with work.

### 3.4. Acknowledged Receiving Support of Husband/Partner during Last Pregnancy

As in 2018, almost all the women interviewed (99.1% in 2019 compared to 97.6% in 2018) acknowledged having received the support of their husband/partner during their last pregnancy, but there was no significant difference by year. Support for medical costs remained the primary type of support received. The main reasons for lack of support in 2018 and 2019 were the husband’s absence or conflicts in the couple’s relationship.

### 3.5. Delivery at Appropriate Health Facilities

Delivery, which is one of the crucial phases of maternity, was carried out mainly at appropriate health facilities with an increased percentage in 2019—85.6% compared to 82.3% in 2018—but the increase was not significant. The clinics, health centres, health huts, health posts, and hospitals were regarded as appropriate health facilities. The main reasons for home delivery in 2018 and 2019 were insufficient time to join a health facility, the lack of transportation means, and the facilities’ remoteness. 

### 3.6. Women’s Empowerment

Women’s empowerment improved significantly. In 2018, only 8.3% of women declared that they made their own health decisions compared to 18.2% in 2019 (*p* < 0.001). More women joined community decision-making associations or groups (30.8% in 2018 versus 53.9% in 2019, *p* < 0.001). Men’s opposition to their wives/partners using contraception (34.4% in 2018 versus 23.7% in 2019) significantly decreased (*p* = 0.001).

## 4. Discussion

This study aimed to confirm the effectiveness of the IEC activities as part of the Plan International Senegal project implemented to reduce maternal and child mortality in the Louga region of Senegal, and to identify related factors that can improve maternal and child health.

Most developing countries in Africa, including Senegal, have strong patriarchal tendencies, thus men exercise absolute control over women and control women’s fundamental health decisions and lifestyles. Therefore, measures to enhance women’s autonomy are actively sought [11,12,13]. For example, under Agenda 2063, which is being promoted by the African Union in relation to the SDGs to further the universal principles of women’s human rights and aim to achieve complete gender equality in all areas of life, efforts have been made to ensure quality education is provided to women [14]. However, despite such efforts, the illiteracy rate of women in Africa remains high at 43.47–86.2% [15,16,17].

In this study, we found most of the men and women interviewed were young and illiterate. Regarding the reduction of maternal mortality, this situation may be cause for concern given the critical role that education can play in raising women’s awareness about the importance of their involvement in decision-making associated with their or their children’s health. The illiteracy rate of women participating in this study was high at 65.2% in 2018 and 53.3% in 2019. According to previous studies, the mother’s education level is an important factor that has a significant influence on the medical service utilisation rate; the higher the education level, the higher the rate of prenatal examination and delivery at facilities [11,12,13,15,17,18]. Mothers with low educational attainment do not receive antenatal examinations, which prevent the early detection of complications that may occur during pregnancy and the opportunity for timely appropriate treatment is missed, resulting in increased disease morbidity [13,19]. A high rate of delivery at home increases the risk of complications due to the inability to cope with unexpected situations such as maternal bleeding and risk of suffocation of the newborn infant during childbirth [19,20]. Therefore, to effectively reduce the maternal mortality rate, efforts need to be made to improve educational opportunities that directly influence women’s use of medical services. However, the customs of child marriage and economic poverty in Africa are reported to be the most significant obstacles limiting women’s educational opportunities [21,22]. Efforts are needed to implement short- and long-term policies to improve them.

An institutional framework for continuous economic growth of the international community in developing countries, abolition of women’s early marriage customs, and a legal framework that makes education above the minimum education level compulsory should be considered under long-term policies. Health education for disadvantaged and poor residents in developing countries will be an effective alternative in the short-term.

The effectiveness of health education that provided information such as reproductive health as well as prenatal and postpartum precautions was studied through the IEC campaign (e.g., conversations, radio shows, home visits, and social mobilisations), and the effectiveness was confirmed by comparing the results before and after the activities. The proportion of men who agreed to the contraceptive method suggested by the wife and to the wife’s autonomy to participate in community associations or groups increased statistically significantly after the IEC campaign.

An effective method to reduce maternal mortality is to increase knowledge of pregnancy danger signs. Knowledge of pregnancy-related danger signs enables the adoption of appropriate preventive and curative behaviours. In this study, after the IEC campaign, men’s knowledge of the signs of danger related to pregnancy and the husband’s companionship during prenatal examinations increased statistically significantly compared to before the campaign. The percentage of respondents who answered that they received support from their husbands during pregnancy did not show any statistical significance but did show an increase compared to before the implementation of IEC activities. In developing countries, the male plays the role of the decision-maker in the household, thus being aware of when to actively support women during pregnancy or reacting sensitively to the women’s abnormal signal is important to the improvement of women’s health by increasing the use of prenatal medical services [23,24,25,26]. Therefore, previous studies are continuing research on methods to increase the participation of spouses and are actively trying to resolve negative factors. In a previous study [23] conducted in Rwanda, when the husband’s participation in pregnancy and childbirth was high, the husband helped with housework or provided medical expenses on the treatment date. However, in cases where the husband’s participation was low, they did not recognise the importance of the antenatal check-up performed before childbirth and did not allow treatment of the spouse.

In a previous study conducted in India [24] to promote male participation in supporting maternal health, the aspects of personal, community, and healthcare were analysed to determine if they acted as facilitating factors or obstacles, and a way to overcome the obstacles was sought. Concerning the personal aspect, the higher the level of education and income, the greater the degree to which the knowledge and awareness of medical services were facilitating factors. It was reported that an obstacle to the participation of men is their system of values, as they value norms and beliefs, attitudes toward gender inequality, and lower participation of women. In the local community, social norms and expectations, and the level of practice of maternal healthcare were found to be factors influencing male participation in women’s maternal health care. The access time, distance, and location of medical facilities, government plans, and programs were healthcare factors that influenced male participation. Daniele [25] found that the level of male participation was determined by various factors. The health care system and its attitude, knowledge, and skills of health care workers regarding prenatal examination and childbirth act as supply factors, and regional attitudes, norms, and male and female preferences change their interaction as demand factors based on the theory of change. That is, if there are no restrictions placed on men in medical facilities, the medical staff view men’s participation positively, and the accompaniment of the spouse for prenatal examinations and childbirth does not go against traditional norms, the level of men’s participation increases. In addition, some women are ashamed of the process of prenatal examination and childbirth being carried out by male medical staff, and it was found that they prefer female companions because they do not want to show their spouses the exam process, are shy, and believe they should endure the whole process of pregnancy alone. Such narrow thinking about women’s gender roles can act as a powerful factor inhibiting men’s participation. It is necessary to educate women so they understand that pregnancy and childbirth are not an experience to be ashamed of, but are extremely important affairs to all families, to society, and to the country.

Many factors that inhibit male prenatal participation may be acting in Senegal; thus, multiple approaches are needed to improve this. The low educational level of men has been reported to be associated with greater adherence to cultural traditions and beliefs [9]. Improving this situation requires the provision of compulsory formal education in addition to the provision of health education through media, which has proven to be effective in improving knowledge of prenatal care [27]. From a community point of view, one method to increase male participation in ANC is to have the village’s authoritative leader recommend that women villagers attend ANC visits with a male spouse as a formal community rule. In areas where ambulances do not have access due to a lack of roads, providing villagers with practical means of transportation such as bicycles or donkey carts, according to the characteristics of the terrain, would enable villagers not only to cope with maternal emergency situations but also to receive appropriate ANC [8]. Lastly, in the case of men accompanying their wives to a medical facility, in many cases they are required to remain in the waiting area and choose an isolated place outside the medical facility until the treatment is completed [28]. In addition, the attitude of medical staff who fail to encourage male participation in the ANC process is perceived as a negative experience, which reduces active participation among men [24,25]. Therefore, facilities should encourage and educate men to get involved in the entire process of prenatal care and childbirth. In addition, for women with limited social relationships, the space for receiving prenatal care needs to be not just a physical place but a space in which they can form social relationships with other pregnant women and share reproductive health information. Therefore, changes in social norms are necessary to enhance women’s autonomy and to recognise that the medical waiting space is a space that includes men, not just women [28].

Although it did not show a statistically significant value, the response rate for childbirth at appropriate facilities did show an increase. According to previous studies, education promotes self-esteem and self-awareness that enable autonomous decision-making [15,29,30]. Women’s autonomy is the ability to make personal decisions regardless of their spouse’s preferences in the home [31], which means improved access to potentially life-saving services for themselves and their children [15], leading to increased use of medical services [13,15,29,30,31]. In addition, women with higher levels of autonomy do not think their rights belong to their spouses but instead believe in a relationship of equality; thus, these women are reported to have implemented family planning using contraceptive methods through effective communication [18,32]. The team of advocates trained by the Korea International Cooperation Agency (KOICA) project promotes awareness through various avenues (conversations, radio shows, home visits, and social mobilisations), urging the population to undergo delivery procedures at appropriate health facilities, for which targets have been achieved or exceeded. However, efforts still need to be made to avoid home deliveries and further improve the condition of facilities to ensure they are welcoming and efficiently care for patients. Most medical facilities in developing countries are not fully functional in terms of infrastructure and resources. In the case of rural areas, there is a lack of transportation and reaching a facility to receive medical services requires extensive travel. In addition, medicines and materials at medical institutions may be insufficient, and in some cases, medical staff are not villagers and may be unprofessional and unfriendly to caregivers and patients [25,26]. Regardless, regular ANC visits and safe delivery, which are basic rights to ensure the mother’s health during pregnancy, should be guaranteed. Therefore, educating women about their rights and national efforts to build systematic medical facilities should be planned and implemented. Improvement of road infrastructure and availability of emergency transport in communes to facilitate the evacuation of women to health facilities are also needed.

In this study, women’s empowerment encouraged them to make decisions about health care, join community activities, and use contraception. In 1994, the International Conference on Population and Development (ICPD) acknowledged that ‘the goal of the empowerment and autonomy of women and the improvement of their political, social, economic and health status is a highly important end in itself and is essential for the achievement of sustainable development’ [33]. Bodily autonomy includes the power to make decisions on health care and contraception, and to decline sexual advances. In Western Africa, where Senegal is located, the levels of power to decide on contraception and health care are 88% and 59%, respectively, which are relatively low compared to the world averages of 91% and 75%, respectively. Additionally, Senegal shows lower percentages compared to the average of Western African countries, 85% and 31%, respectively; moreover, the district in this study, in the lower Senegal regions, show even lowers percentages of 76.3% and 18.2%, even after accounting for the better statistics of 2019. According to the ecological model of bodily autonomy, determinants of women’s decision-making power include gender norms, stigma, and beliefs (community); proximity, cost, quality of care, and provider bias (health systems); the position of partners, communication, and extended family (interpersonal); and education, wealth, media access, and rural/urban area (socioeconomic) [34]. To improve women’s bodily autonomy and achieve empowerment in Senegal, individuals, families, communities, and the state should operate various policies and programs taking these factors into account.

This project-based evaluation study did not design a control group and used a repeated cross-sectional design. This was a limitation of the study. Another limitation was the inability to evaluate effectiveness based on the intervention dosage. Considering these limitations for further studies, it is recommended that evidence is provided by designing a control group and monitoring intervention dosage in a more systematic and scientifically rigorous way.

## 5. Conclusions

In conclusion, short-term education through the IEC campaign in Louga, resulted in improved knowledge of pregnancy among men, more accompaniment of women by husbands/partners, and improvement of women’s empowerment. The IEC activities provide an opportunity to handle the obstacles that occur during prenatal care. The results of this study are applicable to other regions of Senegal and West Africa, but caution is required in East Africa, where religion, climate, society, culture, and healthcare delivery systems are different among the countries of Sub-Saharan Africa. In future, a community-based integrated project to improve women’s health and reduce maternal mortality in Senegal is recommended.

## Figures and Tables

**Table 1:** Characteristics of the participants (women).

		Year				X^2^/t
		2018		2019		*p*
		*n*	%	*n*	%	
Age	<25 years	88	34.8%	261	39.0%	1.831 (0.767)
	25–29 years	62	24.5%	144	21.5%	
	30–34 years	53	20.9%	141	21.0%	
	35–39 years	33	13.0%	79	11.8%	
	40–49 years	17	6.7%	45	6.7%	
Age	Mean ± SD	22.78	6.80	27.49	7.01	0.330 (0.566)
Ethnic group	Ouolof/Lebou	141	55.7%	339	50.6%	7.687 (0.262)
	Poular	101	39.9%	314	46.9%	
	Serere	2	0.8%	3	0.4%	
	Moor	7	2.8%	12	1.8%	
	Others	2	0.8%	1	0.1%	
Education level	Out-of-school	165	65.2%	357	53.3%	13.282 (0.010)
	Koranic	34	13.4%	152	22.7%	
	Primary	40	15.8%	113	16.9%	
	Secondary	13	5.1%	45	6.7%	
	University	1	0.4%	3	0.4%	
Marriage status	Married (monogamous)	149	58.9%	418	62.4%	1.915 (0.384)
	Married (polygamous)	99	39.1%	245	36.6%	
	Others	5	2.0%	7	1.0%	
Religion	Muslim	253	100.0%	669	99.9%	0.378 (0.539)
	Christian	0	0.0%	1	0.1%	
Number of children	Mean ± SD	3.61	2.25	3.41	2.20	1.461 (0.227)
Level of proximity of health facility	Very close	39	15.4%	69	10.3%	11.921 (0.008)
	Close	90	35.6%	278	41.5%	
	Distant	62	24.5%	203	30.3%	
	Very distant	62	24.5%	120	17.9%	

X^2^/t: results of chi-squares/*t*-test.

**Table 2:** Characteristics of the participants (men).

		Year				X^2^/t
		2018		2019		*p*
		*n*	%	*n*	%	
Survey district	Keur Momar Sarr	60	26.7%	223	33.2%	3.505 (0.173)
	Coki	79	35.1%	222	33.1%	
	Sakal	86	38.2%	226	33.7%	
Age	<25 years	4	1.8%	32	4.8%	10.797 (0.056)
	25–29 years	22	9.8%	84	12.5%	
	30–34 years	36	16.0%	114	17.0%	
	35–39 years	49	21.8%	103	15.4%	
	40–49 years	59	26.2%	199	29.7%	
	50 years or more	55	24.4%	139	20.7%	
Age	Mean ± SD	40.76	9.9	40.05	10.40	0.906 (0.365)
Ethnic group	Ouolof/Lebou	109	48.4%	330	49.2%	1.485 (0.829)
	Poular	113	50.2%	325	48.4%	
	Serere	1	0.4%	4	0.6%	
	Moor	2	0.9%	9	1.3%	
	Others	0	0.0%	3	0.4%	
Education level	Out-of-school	42	18.7%	160	23.8%	13.269 (0.010)
	Koranic	160	71.1%	391	58.3%	
	Primary	16	7.1%	72	10.7%	
	Secondary	6	2.7%	40	6.0%	
	University	1	0.4%	8	1.2%	
Occupation	Farmer	73	32.4%	251	37.4%	51.501 (<0.001)
	Livestock breeder	71	31.6%	165	24.6%	
	Liberal professional	47	20.9%	200	29.8%	
	Private sector worker	28	12.4%	14	2.1%	
	Public servant	4	1.8%	26	3.9%	
	Others ^a^	2	0.9%	15	2.2%	
Marriage status	Married (monogamous)	160	71.1%	500	74.5%	1.161 (0.560)
	Married (polygamous)	64	28.4%	167	24.9%	
	Others ^b^	1	0.4%	4	0.6%	
Religion	Muslim	225	100.0%	670	99.9%	0.336 (0.562)
	Christian	0	0.0%	1	0.1%	
Number of children	Mean ± SD	4.98	3.47	4.95	3.56	0.131 (0.895)
Level of proximity of health facility	Very close	29	12.9%	105	15.6%	7.783 (0.051)
	Close	101	44.9%	234	34.9%	
	Distant	49	21.8%	186	27.7%	
	Very distant	46	20.4%	146	21.8%	

^a^: emigrant, ^b^: divorced/separated, widower/widowed; X^2^/t: results of chi-squares/*t*-test.

**Table 3:** The effects of maternal health improvement project focusing on IEC activities.

				2018		2019		X^2^ (*p)*
				*n*	%	*n*	%	
Awareness of danger signs of pregnancy		Males	<3 signs	193	85.8%	531	79.1%	4.793 (0.029)
			≥3 signs	32	14.2%	140	20.9%	
		Females	<3 signs	137	54.2%	392	58.5%	1.425 (0.233)
			≥3 signs	116	45.8%	278	41.5%	
Accompany their wives during ANC visits (female)			No	215	85.3%	517	77.7%	6.508 (0.010)
			Yes	37	14.7%	148	22.3%	
Acknowledged having received the support of their husband/partner during their last pregnancy (female)			No	6	2.4%	6	0.9%	3.118 (0.077)
			Yes	247	97.6%	664	99.1%	
Delivery appropriate health facilities (female)			No	44	17.7%	96	14.4%	1.461 (0.227)
			Yes	205	82.3%	569	85.6%	
Women’s empowerment	Making own health decisions		Only by wife	21	8.3%	122	18.2%	21.935 (<0.001)
			Other than wife ^a^	232	91.7%	548	81.8%	
	Joining a community decision-making association or group		No	175	69.2%	309	46.1%	39.125 (<0.001)
			Yes	78	30.8%	361	53.9%	
	Disagreement about using a method of contraception		No	166	65.6%	511	76.3%	10.667 (0.001)
			Yes	87	34.4%	159	23.7%	

^a^: By both husband and wife, husband/partner, mother-in-law.

## Data Availability

All databases are available from the corresponding author upon request.
